# Embracing the Future Internet of Things †

**DOI:** 10.3390/s19020351

**Published:** 2019-01-16

**Authors:** Flavio Cirillo, Fang-Jing Wu, Gürkan Solmaz, Ernö Kovacs

**Affiliations:** 1 NEC Laboratories Europe, 69115 Heidelberg, Germany; ernoe.kovacs@neclab.eu; 2 Department of Electrical Engineering and Information Technology, University of Naples Federico II, 80138 Naples, Italy; 3 Department of Electrical Engineering and Information Technology, TU Dortmund University, 44227 Dortmund, Germany

**Keywords:** Internet-of-Things, smart cities, hyper-connected IoT, context management, linked data, semantic interoperability, knowledge graph

## Abstract

All of the objects in the real world are envisioned to be connected and/or represented, through an infrastructure layer, in the virtual world of the Internet, becoming Things with status information. Services are then using the available data from this Internet-of-Things (IoT) for various social and economical benefits which explain its extreme broad usage in very heterogeneous fields. Domain administrations of diverse areas of application developed and deployed their own IoT systems and services following disparate standards and architecture approaches that created a fragmentation of things, infrastructures and services in vertical IoT silos. Coordination and cooperation among IoT systems are the keys to build “smarter” IoT services boosting the benefits magnitude. This article analyses the technical trends of the future IoT world based on the current limitations of the IoT systems and the capability requirements. We propose a *hyper-connected IoT framework* in which “things” are connected to multiple interdependent services and describe how this framework enables the development of future applications. Moreover, we discuss the major limitations in today’s IoT and highlight the required capabilities in the future. We illustrate this global vision with the help of two concrete instances of the hyper-connected IoT in smart cities and autonomous driving scenarios. Finally, we analyse the trends in the number of connected “things” and point out open issues and future challenges. The proposed hyper-connected IoT framework is meant to scale the benefits of IoT from local to global.

## 1. Introduction

The Internet-of-Things (IoT) concept has been broadly adopted by heterogeneous communities due to its potential benefits. Progresses in technologies enabled the realization of today’s IoT services. Nevertheless, new technical capabilities are needed to realize “smarter” IoT services or even open possibilities not viable today.

In the current IoT, multiple data sources contribute sensing information and the sensed data is gathered into a single cloud service. Let us consider the example use case of environmental monitoring for noise pollution measurement in a city. Data from sources such as noise sensors and people’s comments from social media are gathered in for the particular cloud service and these sources are used only for the city noise measurement purpose. In the future, on the other hand, produced data need to be shared among multiple applications. Therefore, the linkage between objects, devices, edge devices, actuators, agencies, and services needs to evolve to many-to-many instead of the current many-to-one or on-to-one linkages. This unique characteristic requires transparent discovery and information exchanges opening to more opportunities for information mash-ups among interdependent and symbiotic cloud services.

This article points out the technical deficiencies in the current IoT and proposes a *hyper-connected IoT framework* which connects in synergy multiple interdependent services. The first technical capability lacking in the present systems is the ability to orchestrate data analytics among distributed and federated IoT systems [1], where the orchestration is automatically considering as parameters privacy rules, geographical IoT components topology [2], real-time constraints and Quality-of-Service. A second aspect that hampers the global IoT interconnection is the scarcity of information transparency that encompasses the semantic interoperability capabilities, which ensures consistent data exchange among heterogeneous systems, and the semantic mediation between IoT systems that enables interaction between different system interfaces. Finally, a third aspect is the management of IoT resources in a contextualized manner, in order to overcome the isolation of vertical IoT silos. It is necessary to seamlessly manage diversified information, from physical sensing data (such as temperature observations measured by a sensor on a street lamp post) to high-level contextual information (e.g., disaster detection and monitoring within a geographic area) inferred from the status of multiple things (both physical, and virtual such as social media data). Thus, to realize this vision IoT advancements on data analytics, standardization, and resource and context management are required. While this article focuses on smart cities as important realization of IoT services, the future IoT framework is envisioned for many different applications of IoT such as smart transportation, health-care, public services, smart commercial buildings, smart homes, and smart industry.

As extension of our previous work [3], this article presents also two instances of hyper-connected IoT framework in smart cities and autonomous driving scenarios, both with a detailed depiction of their architecture and functionalities. In addition, we have made a deeper analysis of core concepts of the future IoT such as information transparency, where we have brought an actual example of semantic approach to context managing, and resource and context management, showing a seamless and transparent federation of different heterogeneous systems. We have then performed experiments assessing the performance impact of the federation of IoT systems in comparison with centralized IoT platform. Moreover, we have extended the open challenges discussion including the current advancements into standardization bodies and industrial associations that aim to address aspects of the challenges.

The remainder of this paper is formed by the presentation of the future IoT framework and its technical requirements (Section 2) together with an example of the envisioned IoT framework into an autonomous driving in large scale. Highlighted needed capabilities are deeper discussed in Section 3. Realizations of hyper-connected IoT frameworks are depicted in Section 4. Section 5 reports our evaluation of federated IoT platforms. We then present considerations about trends and growth of IoT and we discuss future challenges and open issues of the future IoT (Section 6). The interested reader might enjoy following the references reported in Section 7. In the last section (Section 8), we make a back overview of the discussed topics and present our next steps on moving towards the future of Internet-of-Things.

## 2. Future Hyper-Connected IoT Framework

### 2.1. Future IoT Framework

**Figure 1:**
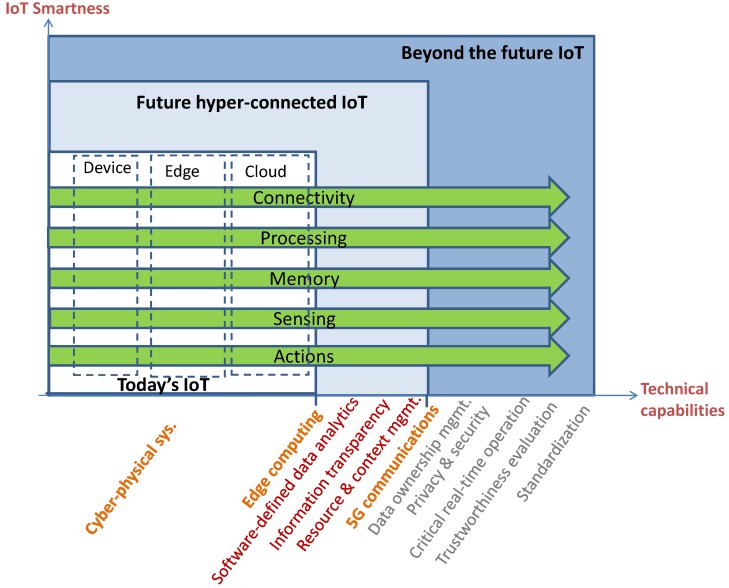
Key technical tipping points towards the future hyper-connected Internet-of-Things.

In this work, we present instantiations and technical capability advancements of the *hyper-connected IoT framework*, proposed in our previous work [3], that envisions the collaboration of multiple services. These services utilize and produce resources (such as data sources, objects, and data analytics components) and context with each other following an omnidirectional topology, differentiating from the vertical approach of today’s IoT instances. The information in the future IoT framework is represented as a knowledge graph shared among multiple IoT domains with both nodes (representing things) and links between them discovered by automated services. Figure 1 summarizes the needed technical capabilities with some of them highlighted (in bold in the figure) that are later discussed in Section 3.

The future IoT framework capabilities are horizontally placed on vertical IoT elements such as device, edge and cloud. Data is produced by sources through the observation of the real-world such as physical phenomena measurements. The capabilities are then fulfilled by functions that are executed either in the cloud or in the edge depending on the hardware resource requirements (such as storage, communication and computation), real-time constraints, and/or optimization of network bandwidth [1]. Moreover, due to the geographically distributed nature of the IoT services, edge computing has become very significant [2]. Some of the data sources might be devices comprehensive of computation and communication capability (e.g., smartphones, cars) hence able to actively participate at the execution of the IoT framework capabilities as dew computing [4].

**Figure 2:**
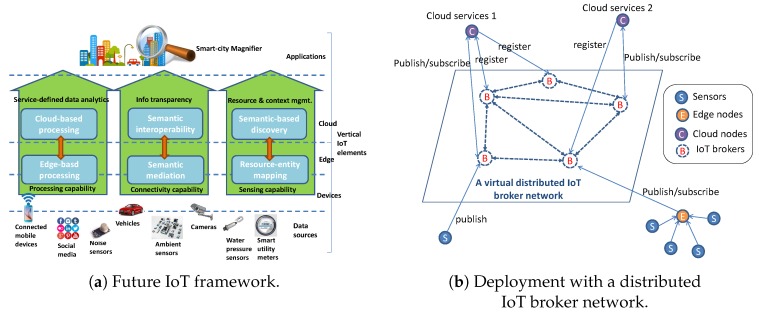
Future Hyper-connected IoT.

Figure 2a depicts the horizontal placement of the highlighted IoT platform capabilities on the vertical IoT elements. The first is *service-defined data analytics* that configures data analytics as a topology of functions, each of which are placed on the cloud or edge (or even device) depending on the requirements of the service. For example, at the edge, some stream analytics are executed in order to reduce the amount of data or to apply privacy-preservation algorithm (such as data anonymization). The cloud, which is orders of magnitude richer in terms of physical resources, is then responsible for performing high-demanding computation for data analytics (e.g., MapReduce tasks). The second capability is the *information transparency* that aims to make the data understandable and usable by heterogeneous IoT systems and applications by hiding the complexity of the IoT elements topology. The translation of raw data to a common form is made by the semantic mediation, usually left to the edge of the IoT (e.g., IoT gateways), whereas the semantic interoperability is performed as cloud services. Finally, the third capability is the *resource and context management* that allows the handling of data not only as isolated pieces of data or isolated datasets but as a global virtual graph of things, having attributes representing the things’ status, linked with each other. The edge is left to associate the incoming real-world observations to a thing uniquely identified by a knowledge base. The data is then semantically discoverable at the cloud level, having the latter a broader view of the IoT elements, making possible a mash-up of heterogeneous information coming from heterogeneous data sources.

A generic future IoT framework deployment is depicted in Figure 2b where each one of the cloud services, often indicated as IoT domains, are handling sensors, edge nodes and cloud nodes. Those cloud services are connected with each other through a network of IoT Brokers that broker IoT messages (requests and data) from a cloud service to another. In order to announce their availability, each of the cloud services announces what their capabilities are through a registration to its IoT Broker.

### 2.2. IoT Framework for Autonomous Driving on a Large Scale

As an application scenario of the future hyper-connected IoT, we analyse an IoT framework for autonomous driving in large scale environments such as cities. We first describe a layered view of IoT for autonomous driving functionalities and then provide a federation view for scalability.

**Figure 3:**
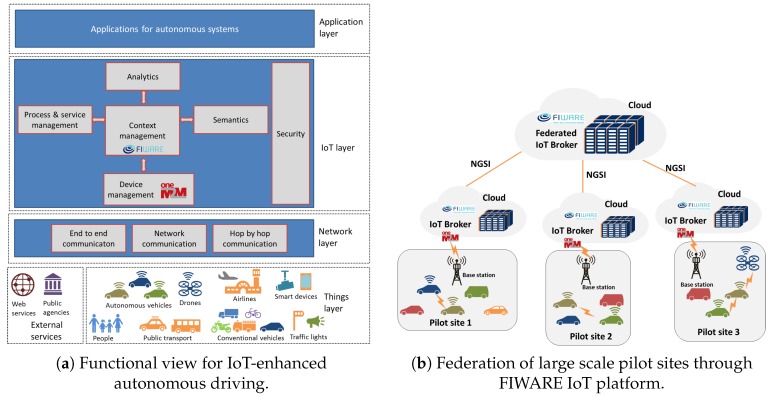
IoT-enhanced autonomous driving. [10]

Figure 3a shows a functional view of a high-level IoT architecture for the considered use cases. This view can be seen as an extension to the IoT Architecture Reference Model (IoT ARM) [5]. There exist four layers, starting from the *things and external services layer* and continues with *network layer*, *IoT layer*, and *application layer*.

The things layer includes any possible actively or passively connected (e.g., observer, actuator, or an object being observed) things. The vehicles include autonomous cars, drones, public transportation vehicles, non-autonomous vehicles, and so on. External services can provide information related to traffic situations or other related information such as weather conditions. The network layer provides the communication capabilities to provide connectivity through advanced 5G technologies and ITS-G5 (Intelligent Transport Systems operating in the 5 GHz frequency band) for vehicle to vehicle or vehicle to road-side communication. The IoT layer has functional building blocks such as device management, context management, semantic interoperability, process and service management, security, and data analytics. Applications of autonomous driving systems are built on top of the IoT infrastructure and they may include services such as optimization of autonomous transportation using cars or taxis.

Figure 3b shows the federated architecture of multiple pilot sites (regions) where each pilot has its own cloud infrastructure served by IoT platforms such as FIWARE [6,7] and oneM2M [8]. The pilot sites are connected to each other through the federated IoT platform. The interfacing between the different pilot sites (or different clouds of pilot sites) are provided by the IoT Broker components which implement Next Generation Service Interface (NGSI) protocol [9].

## 3. IoT Platform Capabilities

### 3.1. Service-Defined Data Analytics

Data analytics in the future IoT needs to adapt to the intrinsic distributed nature of IoT. Thus, the data is processed at every layer of IoT (i.e., device, edge and cloud). This is not only for scalability reasons (such as minimizing network bandwidth by filtering or aggregate useless and redundant data) but also for taking into consideration the high dynamism of IoT infrastructure topology (e.g., new devices joining the IoT system, mobile devices changing their physical location, battery powered devices temporary unavailable because of recharging, intermittent communication channel to devices). Moreover, privacy requirements steer the decision about the allocation of processing tasks since it is always wiser to execute privacy preserving data processing as closest as possible to the generation of the data. The needed analytics are leveraged by IoT services with heterogeneous requirements such as geographical scope of data analytics [2] or Quality-of-Service [11]. The data analytics framework of the future IoT transforms data analytics requests to multiple atomic tasks to be instantiated and orchestrated among the edge and cloud layers depending on the service requirements.

As an example of a service-defined data analytics, we consider crowd mobility analytics [12] that estimates the crowd levels within an area and the flow of people moving between areas. Data sources (e.g., Wi-Fi sniffers, bluetooth beacons, ambient sensors such as temperature sensors) generate observation of the real world. Edge nodes act as gateways, collecting the observations and then sending them to cloud nodes. With the concept of service-defined data analytics, the processing is decomposed in multiple tasks. Thus, the needed analytics can be divided into two categories, the first for lightweight tasks that can run on the edge nodes and the second for power-consuming tasks to be executed on cloud nodes. In the first category, for our example, we can include the *stream processing* part which comprehends a filtering task that filters redundant or unnecessary data, and a privacy preserving task that anonymizes the observations (important for Wi-Fi monitoring). In the second category, continuing the example, we can include the *batch processing* part where historical data is aggregated and patterns are detected.

### 3.2. Information Transparency

The capability of having transparent exchange is fundamental for the future hyper-connected IoT systems. This capability consists of semantic interoperability and semantic mediation. Interoperability is the ability of multiple systems to exchange data. *Semantic interoperability* ensures consistency of data across interacting systems regardless of their individual data formats as these systems attribute the same meaning to the exchanged data. The semantics of data can be defined in a way that different data formats use a shared vocabulary and the vocabulary is specified in an ontology. The concept of semantic interoperability is applicable to all elements of the future IoT framework from data sources to cloud and applications.

*Semantic mediation* is the capability of transforming data coming from one system to make it useful to another system. To achieve interoperability cloud needs to have certain standards so that it can provide contextualized access to the applications. Conversion of data that has certain standards such as oneM2M [13] to other standards such as FIWARE OMA NGSI (Open Mobile Alliance Next Generation Service Interface) [9] can be done on both edge and cloud [14]. Considering that there are many existing standards and communication protocols, semantic mediation is necessary for the realization of communications interoperability between different IoT elements and devices with heterogeneous communication protocols (e.g., Wi-Fi, Zigbee, Bluetooth, 3GPP).

**Figure 4:**
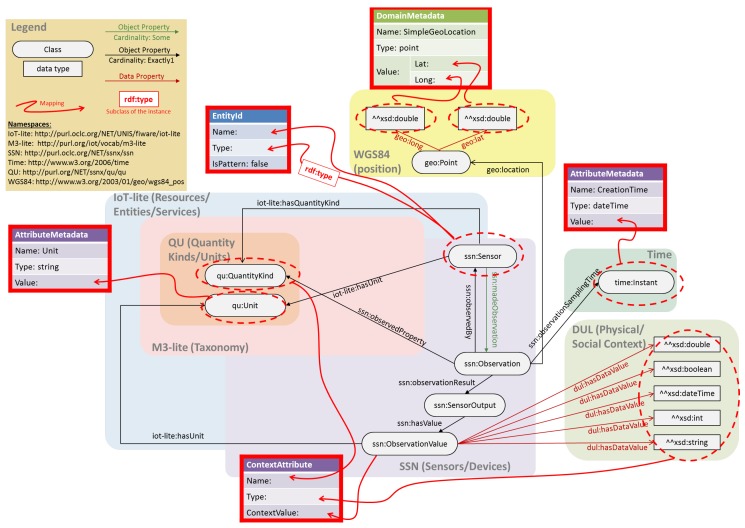
Combining the context management interface standard OMA NGSI with semantics offered by the FIESTA-IoT ontology.

An example of a semantic approach to IoT interoperability is the combination of open standards such as OMA NGSI with the semantic concept of ontology (see Figure 4) such as the one developed within the FIESTA-IoT EU project [15,16] which is a synthesis of known ontologies and taxonomies (such as Semantic Sensor Network-SSN [17], M3-lite [18], IoT-lite [19]) with the aim of covering the contexts generated by multiple heterogeneous IoT deployments. The ontology is conceived to deal with resources description and observations.

In an ontology, rich and complex knowledge is represented as a graphs of nodes, expressing things, and links between nodes that express the relationship between two things. Things are categorized in classes and every thing is an instance of a class. A class may have sub-classes, and an instance of a class is also an instance of all the super-classes of such class. Classes and relationships are specified into taxonomies and an ontology can use classes and relationships from one or more taxonomies. Each class and property used into an ontology is uniquely identified by a namespace prefix and the class or property specific name. For example, *ssn:Sensor* is a class defined in the SSN ontology. For sake of readability, in this paragraph, we are omitting the namespace prefix, but they are all represented in Figure 4. In the FIESTA-IoT ontology, the *Observation* is the core element that bridges the physical phenomena sensed in the M3-lite taxonomy [20] via the *QuantityKind* (e.g., *Temperature*, *CountPeopleMoving*). This taxonomy is fundamental to cover the many different sensors of various IoT deployments. Each *Observation* has a timestamped (*Instant*), a location (*Point*), a unit *Unit*, observed by a sensor (*Sensor*) that is the superclass for all kind of sensors as defined in the M3-lite taxonomy (e.g., *Thermometer*, *PeopleFlowCountSensor*).

In the NGSI standard, each piece of NGSI context data refers to an identifier of the entity, namely *EntityId* and reports several *ContextAttributes*. EntityIds might have one or more *DomainMetadata* which specify metadata in common to all the attributes (e.g., the location of the sensed attributes). In addition, each *ContextAttributes* might have multiple *AttributeMetadata* that are specific to such attribute.

The mapping of the FIESTA-IoT ontology with the NGSI format is shown in Figure 4. The instance of the ssn:Sensor is used as the Name of the EntityId, whereas the specific class of such instance is set as its Type, the *isPattern* (that is used to indicate whether EntityId identifies a range of entities) is set always to false. Each ssn:Observation is uniquely linked to a ContextAttribute that takes the qu:QuantityKind as Name, the class of dul:hasDataValue as Type and the ssn:ObservationValue as ContextValue. Furthermore, each ContextAttribute has one AttributeMetadata for the qu:Unit and another one for the time:Instant. The location of the sensor is stored as a DomainMetadata for all the attributes.

OMA NGSI is currently under study of the Context Information Management (CIM) working group of the European Telecommunications Standards Institute (ETSI) for evolving in a new version that incorporates linked data concepts is and formatted in NGSI-Linked Data (NGSI-LD) [21]. Therefore, it is allowed, if not even recommended, the usage of an ontology in combination with the context management.

### 3.3. Resource and Context Management

**Figure 5:**
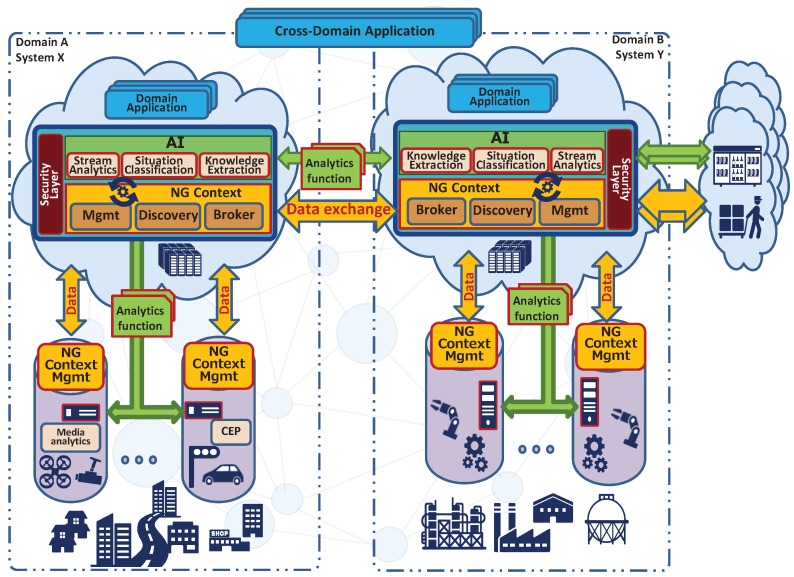
Resources and context management federation.

In the future IoT framework, the resource-context management refers to the *contextualization process* from data to services which requires the capabilities of *resource-entity mapping* and *semantic-based discovery*. In the traditional IoT framework, multiple entities which are objects in the real world (e.g., sensors, actuators, and cloud services) are considered together to provide resources (e.g., such as data and context) for a single purpose. For example, sensing readings from the temperature and humidity sensors in a smart home are only used to trigger the heating system. However, the future IoT platforms consist of multiple interdependent systems that collaborate with each other in a symbiotic manner to share all available resources. As shown in Figure 5, multiple single-purpose IoT deployments (e.g., a smart home, video-surveillance) owned by different parties (e.g., private home owner, homeland security), depicted as silos, have their own sensors and/or devices, computation capabilities to analyse data locally (e.g., Complex Event Processing), and storage capabilities with a context management that handles data. Silos are then handled by a domain administration in the cloud that allows interconnectivity with other domains and therefore other silos. Sharing resources allow, for instance, temperature and humidity readings in smart homes to contribute a city-scale monitoring system. Thus, the capability of resource-entity mapping enables omnidirectional information flows across devices, edge, cloud, and systems to collaboratively leverage these resources. Here, “resources” are not limited to physical sensing data, but they can be high-level contextual information shared among multiple entities in the real world.

The whole interconnection of resources creates a next generation of context, resembling a global distributed graph of information, that can be seamlessly accessed by any actors of the Internet-of-Things. Data requests are brokered by specific components, namely Next Generation Brokers (NG Brokers), that dispatch data requests to providers of the requested data and aggregate the responses before to return back to the requester achieving a transparent global IoT since the IoT topology is kept hidden from data producers and data consumers perspectives.

Semantic-based discovery removes the need for human involvement and assistance and allows worldwide IoT applications to have fully automated reconfiguration and information mash-ups. High-level contextual information are computed by processes of *knowledge extractions* that combine sensed resources mapping them to common entity models and information model. This capability generates new metadata and attributes necessary for linking information into a context mash-ups. For instance, crowdsensed measurements of noise through smartphones [22] are aggregated to assess the noise level of a city neighbourhood level. The produced data and knowledge might be not of much value if those are not interpreted for understanding a situation of the real world. For example, some indexes of high values of temperature and CO2 together with detected hectic crowd patterns, if correctly interpreted, would permit the identification of an ongoing fire break. This interpretation is done by *situation classification* analytics components, of which output is either signalled to a human or automatically triggers actions. In addition to these specific contextualization computation, other *stream analytics* processing are executed for various purposes such as privacy preservation, data polishing, sensor fusion. As we have seen in Section 3.1, we envision the data analytics as topologies of atomic *analytics functions* (or tasks) dynamically instantiated within the *Artificial Intelligence (AI)* layer federated among systems (see Figure 5).

Due to the fact that computation and storage capabilities are distributed, data analytics routines, modelled as topologies of analytics functions (e.g., lambda functions), might be physically distributed according to several optimization directives with different perspectives [1] orchestrated by a stream analytics system. The synergistic work of analytics and data flowing towards the analytics functions is handled by the next generation context management exploiting the common meaning given to the information among the hyper-connected IoT.

Security layer, horizontally placed on all the components handling and exposing data, is managed by local administrations that allow the owner of the IoT deployments to have full control of the data, and consequently regulates analytics access and computation over the data.

## 4. Realization of Hyper-Connected IoT realm

In this section, we are showing two examples of an actual realization of the hyper-connected IoT vision based on two real-world scenarios: a smart city and IoT augmented autonomous driving.

### 4.1. Smart-City Magnifier: Smart Cities Enabled by Future Hyper-Connected IoT

As an example of future application that makes use of cross-cloud interoperation, we have implemented a smart city application, named Smart-City Magnifier. The application purpose is to analyse the situation of generic geographic areas and compute indicators representing the health of the urban and natural environments. The outcome of the indicators are then showed into a dashboard that highlights the critical situations such as emergencies (e.g., fire breaks), high crowd densities, and urban and rural pollution. The data is harvested from data sources belonging to different IoT domains hosted by separated IoT clouds and going through a process of associating observations to real-world things (e.g., a city or a neighbourhood) and then analyse the situations related to those things.

**Figure 6:**
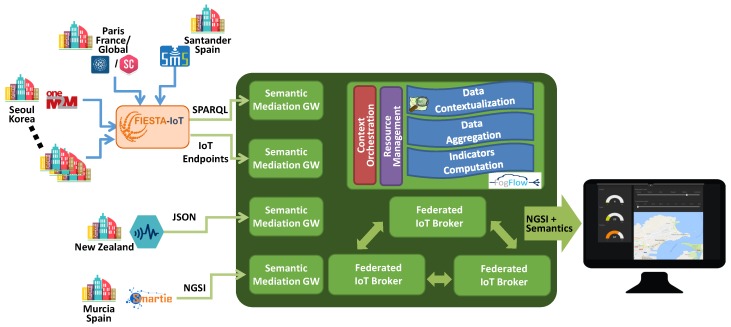
Realization of the Smart-City Magnifier application leveraging *semantic mediation*, *semantic interoperability*, *resource orchestration* and *federation of IoT platforms*.

Figure 6 shows the concrete implementation architecture of the Smart-City Magnifier application. Data are acquired by many Semantic Mediation Gateways (SMGs) that have the purpose of semantically translating from a format (protocol and data model) to another format. In this application, SMGs transform data to NGSI format modelled with FIESTA-IoT ontology (see Section 3.2). The integrated IoT deployments are either coming from the FIESTA-IoT system [23,24], from crowd mobility IoT deployments in New Zealand or from the smart city of Murcia offered by the SMARTIE EU project [25]. The deployments behind the FIESTA-IoT framework are exposing the IoT data either through the FIESTA-IoT historical triple store accessible via SPARQL Protocol and RDF Query Language (SPARQL) queries or via exposed IoT endpoints, discoverable again through a SPARQL query, for every available sensors. In both cases, the data format is plain JSON-Linked Data (JSON-LD) annotated with the FIESTA-IoT ontology. Through the FIESTA-IoT platform, more than 7500 sensors or resources could be accessed from 10 testbeds reporting environmental data (e.g., outdoor temperature, humidity, particles concentration, luminosity, noise level), road traffic monitoring (e.g., vehicle speed, traffic intensity), car and bike parking spots, public transportation status (e.g., bus estimated arrival times, vehicle localization), garbage management, soil and trees monitoring (urban parks and garden, rural areas), pedestrian presence detector, electromagnetic outdoor exposure, smart building/office information (e.g., human presence, power consumption, heating ventilation and air conditioning-HVAC system, solar panels), signal power and power consumption of wireless sensors, and sea water quality (e.g., pH, Ammonium). The crowd mobility deployments from New Zealand are instead exposing the data via an ad hoc JSON format. The Murcia smart city data, comprehensive of 430 city wide urban mobility sensors (e.g., traffic sensors, bike and car parking spot sensors, public transportation status sensors) and 25 smart building sensors in the university campus, can be accessed via the NGSI interface, which is following an ad hoc data format defined by the specific IoT silo owner.

The interoperable data is then, on the one hand, handled on multiple IoT Broker instances federated in a topology of peers, and, on the other hand, orchestrated by the *FogFlow* [1] framework which offers a service-defined analytics layer (see Section 3.1). The federation of brokers is meant both to enhance the scalability of the whole system, since the data coming from the IoT deployments are sharded among them, and to differentiate IoT domains. Any of the brokers is able to provide all the available data in the federation through a single NGSI request, necessitating sometimes of a multi-hop connection, transparently executed, if data are not directly provided by the contacted broker. The data analytics instead is made of atomic tasks handled and instantiated by the FogFlow framework which manages the available computation resources and orchestrates the data stream between tasks. Since the future smart cities take place in a digital real-time world, real-time information fusion through the hyper-connected IoT framework are required to provide contextualized information to the different levels for executing necessary actuations. A first task performs *Data Contextualization* that associates virtual entities to the incoming observations. Contextualizing, in this scope, is the act of inferring the real-world things (e.g., a building, a street, a square, a suburb, a city etc.) to which each geotagged observation belongs, making usage of the knowledge storage offered by external knowledge bases such as OpenStreetMap [26]. One observation might be associated to one or more real-world things, for instance, an observation of outdoor temperature sensor attached to a building wall can be associated to such building, to the street where the building is located, to the neighbourhood and to the city. At the same time, one thing can have more than one observations associated, for example, if two temperature sensors are located on two buildings in the same street, such street has two sources of temperature observations. Since the data contextualization does not need raw data from other sources, it can be executed on the edge or on the cloud specific of the data source domain without accessing the federation. The *Data Aggregation* groups the incoming observations by the inferred things. The aggregation makes usage, for instance, of statistics means. The *Indicators Computation* is calculating smart city Key Performance Indicators (KPIs) [27] assessing the status of city and identifying critical situations. The latter two data analytics tasks necessitates cross-domain data and therefore they make data requests trough the federation. Relating the Smart-City Magnifier with the conceptualization of Figure 5, data contextualization and aggregation tasks are knowledge extraction whereas indicators computation task is situation classification.

The output of the analytics is handled again by the federated IoT Brokers. The dashboard is then acquiring the information and visualizing it on a map presenting the alerts and warnings with the means of several visualization widget tools. The same visualization dashboard is used for visualizing all the situations inferred from the IoT silos integrated in Europe, Korea and New Zealand, with a total of 13 city areas (Guildford in the United Kingdom, Santander and Murcia in Spain, Seoul in South Korea, Toulouse and Paris in France, Heraklion and Volos in Greece, Waterford in Ireland, Minervino Murge in Italy, Wellington and Christchurch in New Zealand). Since the analytics processing tasks are working on homogeneous semantically annotated data, the geographic scope of the dashboard map can simply be navigated to focus on a different city.

### 4.2. Smart Mobility: IoT-Enhanced Autonomous Driving

We built a Crowd Estimation and Mobility Analytics (CEMA) service which is beneficial for enhancing autonomous driving through IoT. Here, we focus on the *crowd size estimation* given by the service based on the level of Wi-Fi activities in the surrounding of the deployed Wi-Fi sniffers. Wi-Fi sniffers are statically deployed on the road-side as well as in the autonomous vehicles by including GPS sensor for knowing the positions of the vehicles.

This subsection describes the use of the proposed CEMA, federation, FogFlow, and SMG technologies in the smart mobility domain for IoT-enhanced autonomous driving. Integration of these technologies with other IoT and/or autonomous driving components makes the use of them possible in the pilot tests, which are conducted at the Technical University of Eindhoven campus in the Netherlands. The purpose is to estimate crowdedness in the university campus and leverage measurements coming from many vehicles to create a dynamic map of the crowd. By using the dynamic map, the autonomous vehicles can make routing decisions such as choosing a less crowded path or other decisions such as speed enforcement during certain events where many pedestrians exist.

**Figure 7:**
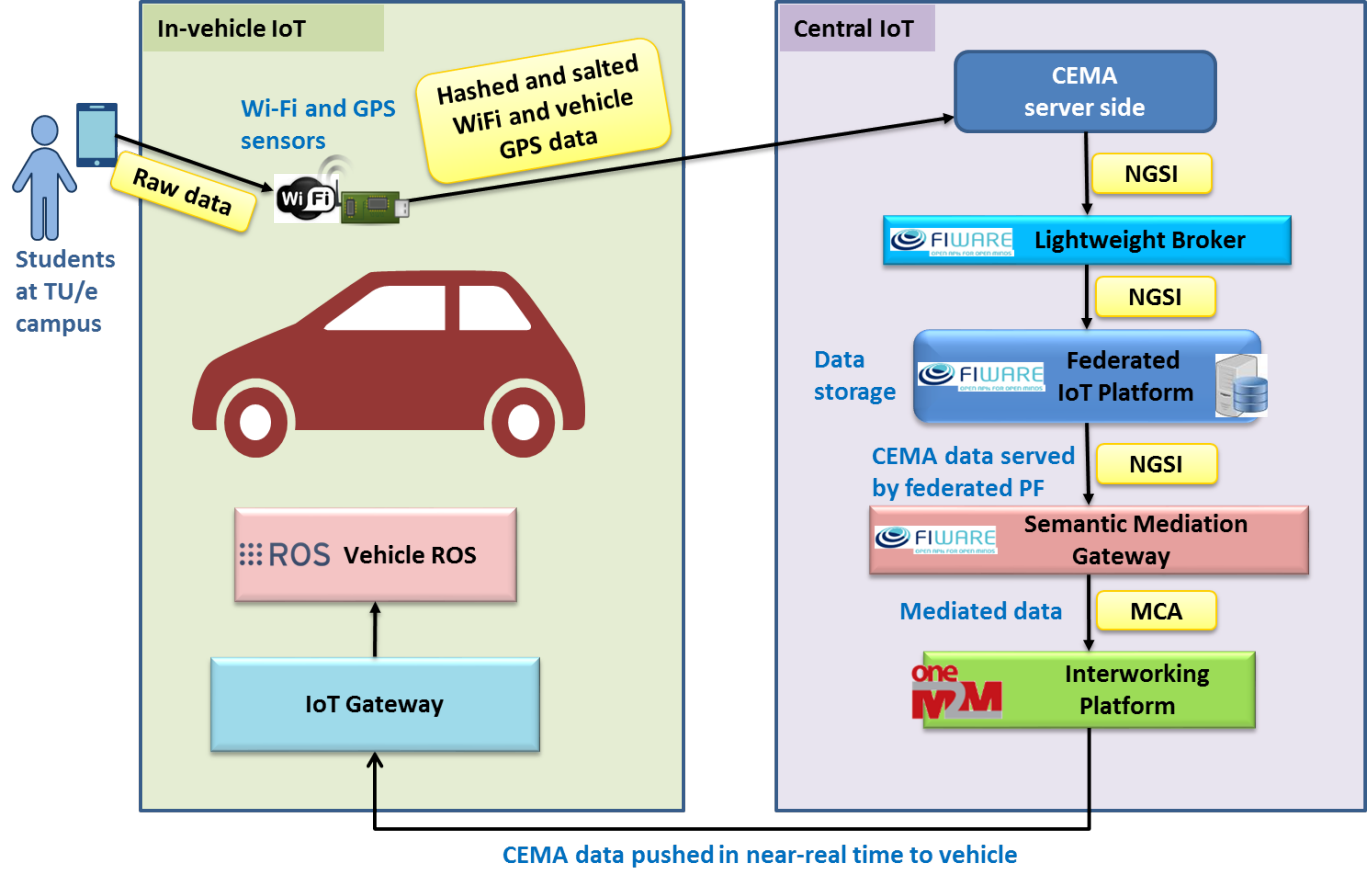
Integrated cloud and edge technologies: from vehicle to vehicle.

Figure 7 shows the integration of components for the aforementioned purpose. In the “In-vehicle IoT” side, we deploy a device including Wi-Fi and GPS sensors in the autonomous driving vehicle for sensing the Wi-Fi activity in its surrounding. The Wi-Fi sensor (constituted of a Wi-Fi module configured in monitor mode, sniffing Wi-Fi probe requests) collects raw data from smartphones of students at the campus (such as MAC address of the requester, relative received signal strength-RSSI, and timestamp) and the device at the vehicle anonymizes the data using hashing and salting mechanisms and pushes to the cloud side where “Central IoT” resides. Crowd estimation service (CEMA) [28] creates the analytics results and makes them available through a lightweight broker for the campus. Several pilot sites can be connected through the Federated IoT Platform. The data in the NGSI format is translated to Mca (reference point for M2M communication with application entities, see [13] for details) by SMG and pushed to the shared oneM2M-based interworking platform. The vehicle subscribes to the oneM2M platform through an IoT gateway physically placed in the vehicle. The gateway pushes the data to the robot operating system (ROS), which has subscriber components such as routing decision components. Moreover, ROS is used for the control of the vehicle itself, so that the decisions, such as speed reduction or alignments, are conducted through ROS. In this manner, the data collected by the autonomous vehicle is pushed back either to the vehicle itself or to other autonomous vehicles.

Instead of performing data analytics on the central IoT, an alternative approach is to migrate whole detection, processing, and analytics tasks toward the front-end devices, only the data analytics results are published to central IoT. In this case, lightweight edge-based crowd mobility analytics modules can be performed in a single device attached inside automated public transport. Similarly, an edge device is integrated with a GPS sensor and an inertial measurement unit (IMU) sensor for performing stop detection, passenger estimation, and passenger flow tracking. On-board processing and analytical approaches are more privacy-preserving and consume less energy for data communications. Experiments are conducted in the automated hanging trains system at Technische Universität Dortmund.

## 5. Evaluation

We have performed experimental tests with the aim of analyzing the impact of a topology of federated IoT platforms instead of a single cloud IoT platform instance. In particular, we have made usage of the FIWARE foundation [7] and its portfolio of components for creating various IoT platform configurations. The first set of tests is meant to understand the impact on the performances when the data requests are not handled by a single component in the cloud but into a federation of platforms. In the former case, the data is directly requested to the provider of the data; in the latter case, for each query, a discovery is performed first to find the provider of the requested data and then the data is provisioned to the requester, hiding the complexity of the system behind. We have used the dockerized reference implementation of the FIWARE Context Broker Generic Enable [29], which is recommended by the European Commission as one of the Connecting European Facility [30], as the centralized IoT platform. On top of it, we have then instantiated a federation framework comprehensive of a broker of IoT messages, using the reference implementation of the FIWARE IoT Broker Generic Enabler [31] together with the alternative implementation of the FIWARE IoT Discovery Generic Enabler [32]. All the components are using the OMA NGSI standard [9]. In the case that the IoT query is handled by the centralized system, the request is issued directly to the Context Broker (CB) that performs a lookup on its local database and responds to the requester. However, the queries handled by the federated framework are sent to the IoT Broker (IoT-B), which discovers providers through the IoT Discovery (IoT-D) component and then requests all the discovered providers (in our case several CBs) with the requested data. The responses are then collected by the IoT-B and sent back within a single message to the requester. The latter is not aware of the complex topology hidden behind the IoT-B.

**Figure 8:**
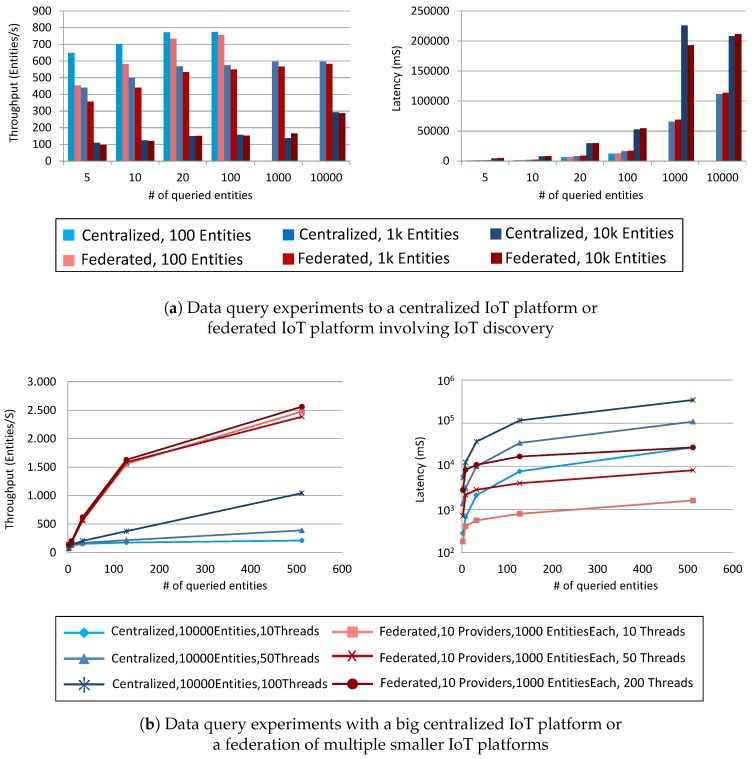
IoT platform experiments: (**a**) performance impact of the federation of IoT platforms; (**b**) scalability of IoT platforms’ federation.

Figure 8a shows the result of performing query to a centralized and federated IoT system. The federation is made of an IoT-B and an IoT-D that are hiding an instance of CB. The plots show on the *x*-axis the number of entities (i.e., things) requested in each query. The throughput is measured as the number of entity data (fixed to 20 attributes per entity) per second. Several sizes of the IoT system are tested: 100, 1000 and 10,000 entities. The requests are made using 100 parallel clients continuously requesting the systems. The plots show that the performances, both throughput and latency, are not affected much by a big volume of data exchanged, although the federated configuration requires more operations and generates more messages.

We have then performed a second test for assessing the scalability advantages implicit of the federation. We have envisioned a scenario of 10,000 entities handled both by the centralized system and by a federation of 10 different providers, each one managing a disjoint set of 1000 entities. The test has been repeated for 10, 50 and 200 parallel clients (i.e., threads), requesting 2, 8, 32, 128 and 512 entities per query. Figure 8b clearly shows that a federation with several small data providers with sharded dataset are outperforming by orders of magnitude the centralized approach.

A federated platform has many advantages, for example: data providers can handle data locally preserving privacy and data sovereignty, a new data provider can join the federation (by performing an NGSI-9 RegisterContext) at any time since a discovery is performed for each request (we are not considering the case of caching), complex semantic discovery can be performed at discovery time, service-defined data analytics can be triggered on demand at the time of the query. In addition, a heterogeneous system can join the federation making usage of mediation gateways for translating the data format and interfaces methods.

## 6. Challenges and Open Issues

We have performed a trend analysis based on the reports from International Data Corporation (IDC) [33], Gartner [34] and our estimations [3]. In addition, 11.4 billion “things” were installed in 2014 and 13.7 billion in 2015 with a 20.8% increase. Even if the numbers are forecast to continue increasing, the rate will gradually decrease to 11.5% in 2020, with an estimation of 28.1 billion ”things”. Consequently, the generated data volume will grow by an order of magnitude (10×) from 2015 to 2020 [35]. This exceptional growth of IoT is due to the promised financial benefits. Smart cities and smart homes are, amongst the others, the most advanced field where many businesses compete to earn a share of the market. In the smart cities field, a big push was made by the governments that gradually increased investments, whereas the change in the way of people thinking about everyday life, always more digitalized, opens great business opportunities in the smart homes area. According to [3,33,34], the market share of smart homes and smart cities is forecast to be the 25% over the total IoT market, by 2020.

These vast numbers of connected “things” and large data volumes are only possible with new technologies and standards. The unprecedented increase in “things” coming together with big data brings many new challenges and problems in connectivity, processing, memory, sensing, and actions that require enabling of the future IoT platform capabilities and 5G in order to lead the aforementioned expectations into a reality. Based on this observation, we point out some open issues in the future IoT as follows:

(1) *Data ownership management*: For a future IoT where data is globally accessible and discoverable, special attention should be paid in order to assure that the producer of the data (or the owner of the observed things) keeps ownership of the data, especially for privacy-sensitive data. A study of the International Data Space Association (IDSA) [36,37], where more than 200 companies have been interviewed regarding data exchanged with other companies, states that one of the major concerns that blocks a company from sharing data with another peer is the uncertainty of losing control over the data once the data has been released, and thus losing the “sovereignty” of the data. A first issue is to state “who is the owner of the data”—for instance, we are keen to think that the owner of the IoT deployment is the owner of the data; for example, a public transportation company deploying sensors on its buses is the owner of such data. However, in other situations, the owner of the data is the observed thing; this is the case of health sensors deployed by the health care system at home of a patient where the patient is the “thing” observed and the owner of the data. In addition, another open issue is “how to control the data migration to other users and services”. Often, users are requested to sign agreements on processing their data, as specified on common data regulations (e.g., General Data Protection Regulation-GDPR [38], but, afterwards, there is not an easy way to control if those agreements are respected. In addition, the data owners should be capable to visualize where, how, by whom and why their data are accessed. Moreover, usage terms might dynamically change over time due to new regulations, changing of the mind of the data owner, or other factors (e.g., expiration of a time period). An automatic system of managing these data access rights’ dynamism is a clear challenge.
(2) *Privacy and security*: With the realization of the presented capabilities, the future IoT will encounter new security and privacy threats. Every IoT layer, from application to devices, has peculiarities on the security risks and possible attacks. Considering the vertical elements in the bottom-up architecture, each level (i.e., devices, edge, cloud and applications) has its own security requirements. Each level is exposed to various types of security threats and possible attacks. Currently, there is a lack of and a certain need for a dynamic IoT security model for enabling mission-critical applications (e.g., autonomous vehicle control) and expected advancements in the IoT systems. Furthermore, for building trust and secure relationships between the IoT components, proper identification and authentication capabilities, and cooperation among these techniques in the IoT platform are currently missing. On the other hand, preserving privacy of data in IoT is an open challenge. The existing privacy protection policies for today’s IoT include *encryption*, *anonymization* and *obfuscation* techniques, which are mainly for single services. However, new privacy preservation techniques in these interdependent services (e.g., searchable encryption, usage control, end-to-end encryption [39] with homomorphic encryption) by design principle for objects, devices, users, subsystems, and services are required.
(3) *Critical real-time operation*: The IoT of the future should be flexible and adaptable to sudden changes of the status and conditions of the infrastructures. This is due in order to have fast response to critical situations such as the increasing frequency of natural disasters due to the global climate change [40]. Infrastructureless alternatives for communication in networks [41] or easy-to-deploy infrastructures [42,43] can help solve these problems.
(4) *Trustworthiness evaluation*: A dual problem of the data access control is the control over data generation. Since the data is associated with real-world things reporting the status of them, only legit data sources should be allowed to report observations to a thing and, at the same time, the future IoT should be resistant to tampering attack. For that reason, it is a challenge to make a trustworthiness evaluation assessing which entity might be trusted and how trustful is the data generated [44].
(5) *Standardization*: Different layers of IoT have been studied within many standardization activities. However, there is little consensus regarding which layers and relevant techniques should be standardized and which layers should remain open to be designed. In addition, governments showed their interest in standardization and their involvement implies innovation restrictions due to ever stricter regulations. New requirements for IoT are defined by IoT organizations such as OpenFog, the Industry 4.0, Made-in-China 2025 [45], and the Industrial Internet Consortium. New activities are expected to come from ETSI, IEEE, IEC, ISO, FIWARE and oneM2M, to name a few. The advancements in standards should cover every ICT field such as connectivity (e.g., 5G and satellite connections), data format and models (e.g., semantic interoperability and data contextualization), sensing, actuations and security at all levels.

A one-size-fit-all network management based on the same physical network infrastructure to exchange a variety of traffic and context is not efficient. Therefore, 3GPP considers a more flexible network slicing technology that is one of the important features in 5G. Currently, the 3GPP SA2 Working Group has defined the following three different categories of network slices based on the characteristics of service requirements: (1) extreme mobile broadband (xMBB), (2) ultra-reliable and low latency communications (uRLLC) or ultra-reliable MTC (machine-type communication), and (3) massive MTC (mMTC). First, high data rates and low latency are expected for the first type of services. Second, a critical level of latency and reliability are expected for the second types of services such as mission critical emergency response services. Third, machine-to-machine and device-to-device wireless communications are expected for the third types of service such as communications between lightweight IoT devices.

## 7. Related Work

The book by Vermesan and Friess [46] analyses extensively the trends and the innovation in many IoT applications fields bringing a good photography of the status of the European IoT research and innovation together with a depiction of challenges and issues to be tackled. In addition, some technical aspects are discussed, such as privacy and security, IoT analytics, and linked data. Similarly, Al-Fuqaha et al. [47] exposes a good overview of the available technology for IoT and the challenges to be addressed.

Technical capabilities exposed in this article are also addressed by specific studies. A work related to service-defined data analytics (see Section 3.1) is proposed by Naranjo et al. [48] that describes a smart city system based on edge computing. It makes usage of edge nodes to execute IoT applications tasks in order to improve the efficiency in terms of energy consumptions and latency. The research efforts from Bonomi et al. [49] and Mahmud [50] systematically describe the field of edge computing for IoT. The unified ontology presented by Agarwal et al. [15], meant for heterogeneous IoT systems, is approaching the information transparency (see Section 3.2) in IoT. The capability of semantic mediation (see Section 3.2) has been realized and has been implemented in the European WISE-IoT project [51]. A Morphing Mediation Gateway [52] enables the interworking between heterogeneous IoT platforms and between IoT devices and IoT platforms. Soldatos et al. [53] introduces a solution for transparently brokering semantic query to federation of systems, relating to the context and resource management (see Section 3.3). One of the few studies regarding the contextualization of IoT data (also in Section 3.3) is presented by Yavari et al. [54].

From the application perspectives, the research efforts of Gerla et al. [55] and Rahim et al. [56] describe the advancements of the IoT in the automotive fields (see Section 2.2 and Section 4.2). Suciu et al. [57] is proposing a platform for enabling collaboration among multiple parties in order to enable an ever increasing ecosystem of services for improving the quality of life within a city and, at the same time, allowing business. Santana et al. [58] presents a unified reference architecture for Smart Cities (see Section 4.1). Memos et al. [59] integrates a Wireless Sensors Network (WSN) for surveillance application into a smart city framework taking into consideration security and privacy concerns. Finally, our previous work [28,60] and the work from Chilipirea et al. [61] treat the topic of crowd analytics using the Wi-Fi signals (see Section 4.2). In the study of Andión et al. [62], a wide review of human activity detection based on Wi-Fi signals is also included.

## 8. Conclusions and Future Work

In this work, we have introduced a vision of the future Internet-of-Things which foresees a global interconnection and interworking of heterogeneous devices, systems and services. Several technical capabilities toward the future IoT laying on device, edge and cloud IoT layers are needed. Some of the key technical tipping points, such as service-defined data analytics, comprehensive of elastic edge-cloud orchestration, information transparency, data contextualization, and semantic discovery, are extensively analysed with the help of two concrete examples of hyper-connected IoT frameworks in the scenarios of smart cities and autonomous driving. The experiment results regarding the federation of IoT platforms show that the overhead for enabling a hyper-connection of systems is much lower than a big amount of data exchanges. In addition, the results demonstrate that the federation allows the scalability of IoT systems when the size of IoT scenarios increases. We have outlined challenges and open issues to be addressed by researchers and industry in the near future.

As the next step for realizing the vision of the hyper-connected IoT, we are adopting the new standard of the ETSI CIM [21], which natively allows IoT linked data. This enables the possibility of semantic reasoning over the data. In addition, we are planning to extensively work on knowledge extraction and situation classification. Exploring the semantic linkage among IoT data and creating new understanding of the real-world and discovering connection between data and things are future promising directions. Finally, we are actively taking into consideration the privacy and security aspects following the International Data Spaces Association (IDSA) directives [63], experimenting with new manners for always leaving data sovereignty to data owners by exploiting and improving the FogFlow framework and programming [1].
